# Supplementary figures and images for: The non-selective Rho-kinase inhibitors Y-27632 and Y-33075 decrease contraction but increase migration in murine and human hepatic stellate cells
Source: PLoS One. 2023 Jan 31;18(1):e0270288. doi: 10.1371/journal.pone.0270288 (PMC9888688; doi:10.1371/journal.pone.0270288)

Figure 1G

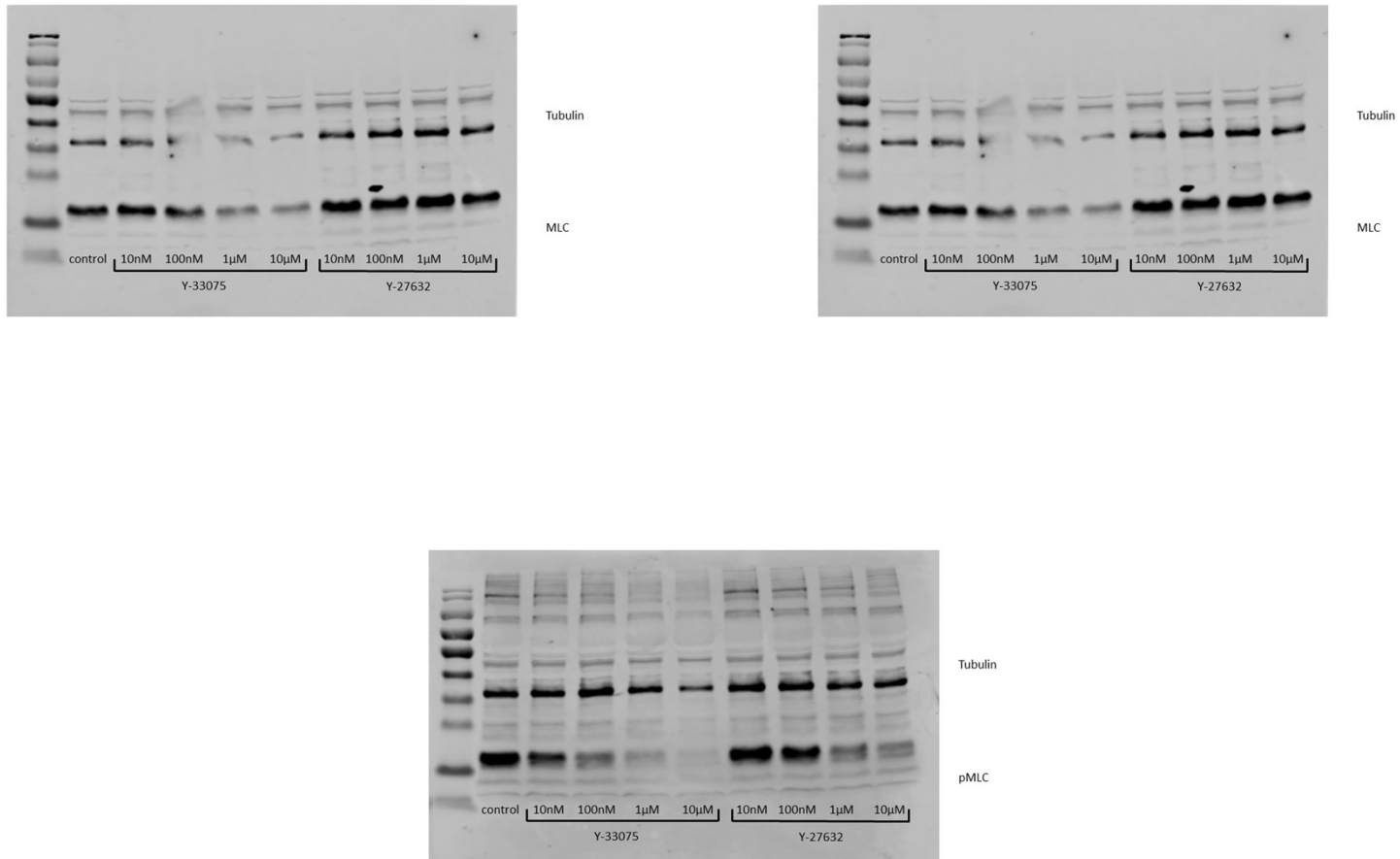

Figure 2A

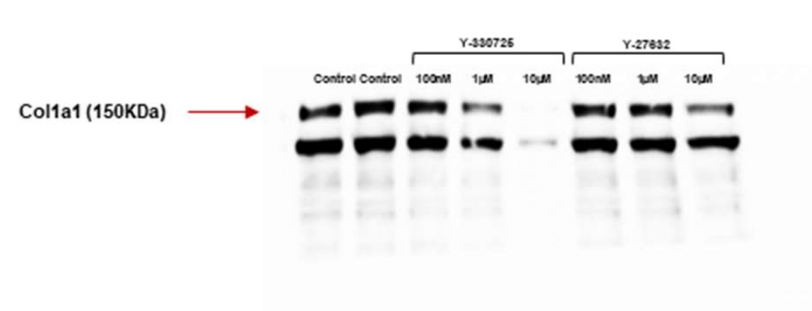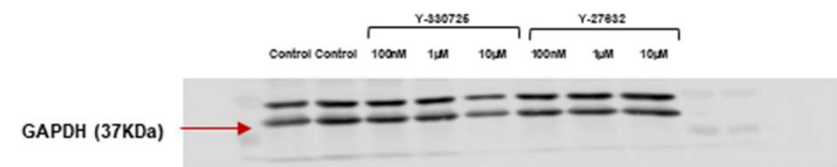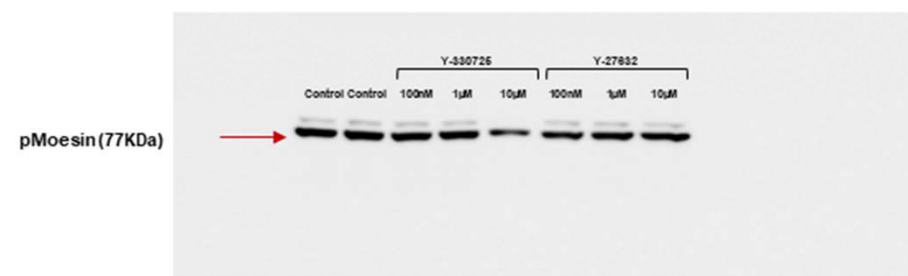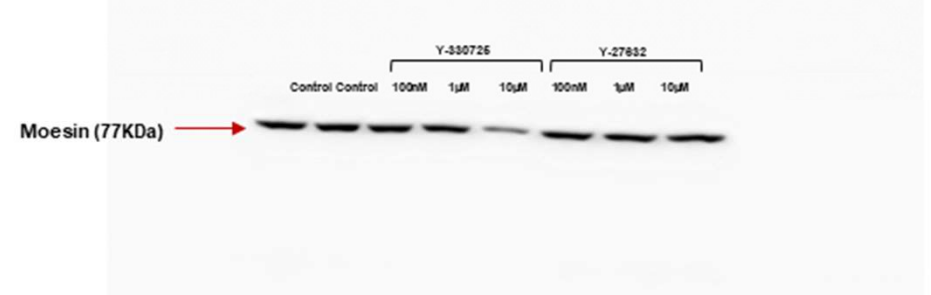

Figure 2C

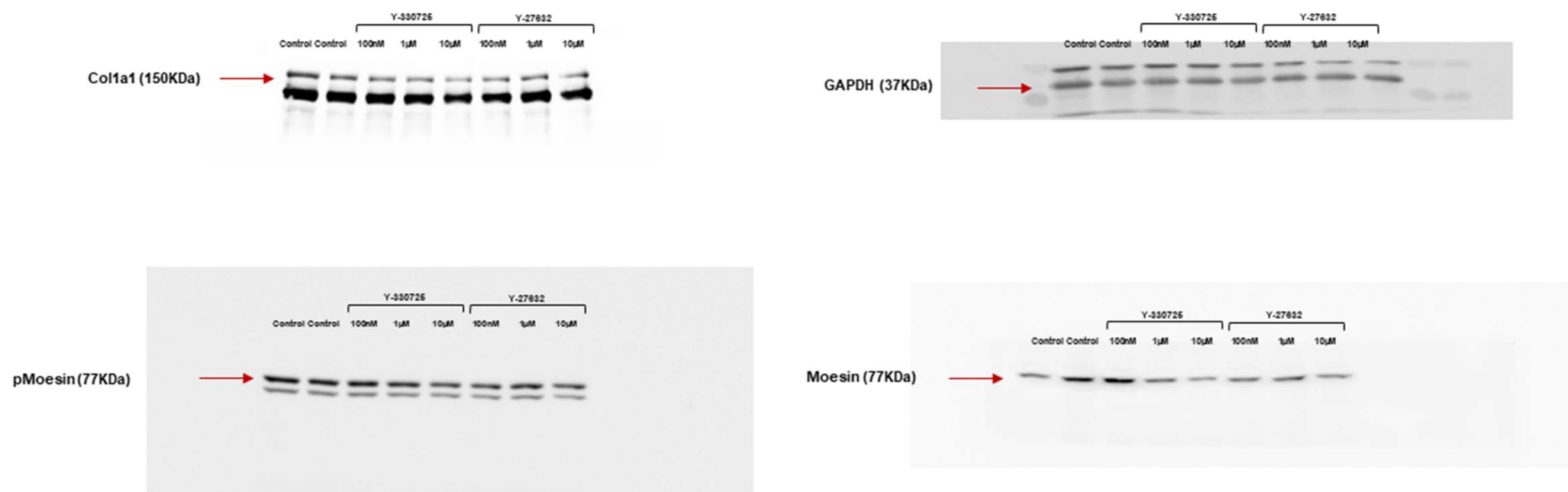

Figure 4E

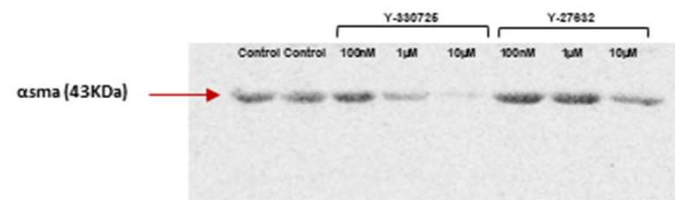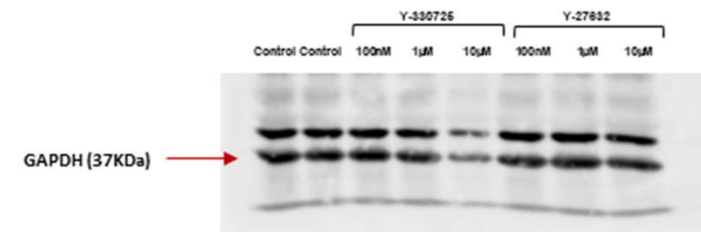

Figure 4G

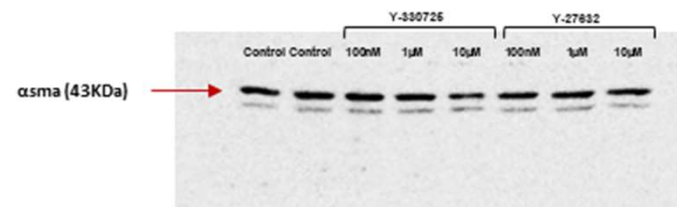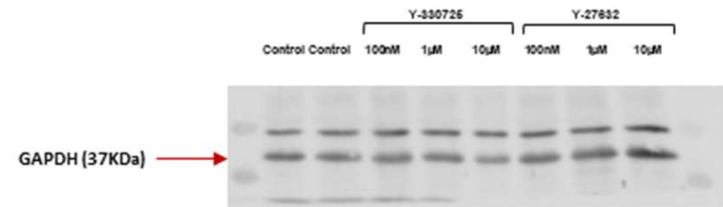

Supplement: S1 File — (PDF) [file pone.0270288.s001.pdf]
