# Supplementary material for: The non-selective Rho-kinase inhibitors Y-27632 and Y-33075 decrease contraction but increase migration in murine and human hepatic stellate cells
Source: PLoS One. 2023 Jan 31;18(1):e0270288. doi: 10.1371/journal.pone.0270288 (PMC9888688; doi:10.1371/journal.pone.0270288)
Supplement: S1 Table — (DOCX) [file pone.0270288.s002.docx]

**S1 Table. Antibodies.**

| **Name** | **Order Number** | **Company** |
| --- | --- | --- |
| α-SMA | [ab5694](http://www.abcam.com/alpha-smooth-muscle-Actin-antibody-ab5694.html)-100 | Abcam plc, Cambridge, UK |
| Col1a1 | 1310-01 | Southern Biotech, Birmingham, AL, USA |
| GAPDH | sc-47724 | Santa Cruz Biotechnology, Santa Cruz, CA, USA |
| pMoesin | sc-12895 | Santa Cruz Biotechnology, Santa Cruz, CA, USA |
| pMLC | 8505 | Cell Signaling Technology, Inc., MA, USA |
| MLC | 3674 | Cell Signaling Technology, Inc., MA, USA |
| α -Tubulin | CP06 | Calbiochem, San Diego, CA, USA |
| a-Mo IgG HRP | ab97265 | Abcam plc, Cambridge, UK |
| a-Go IgG HRP | sc-2768 | Santa Cruz Biotechnology, Santa Cruz, CA, USA |
| a-Rb IgG HRP | 7074S | Cell Signaling Technology, Inc., MA, USA |
